# A dose-ranging, parallel group, split-face, single-blind phase II study of light emitting diode-red light (LED-RL) for skin scarring prevention: study protocol for a randomized controlled trial

**DOI:** 10.1186/s13063-019-3546-6

**Published:** 2019-07-15

**Authors:** Julie K. Nguyen, Jeremy Weedon, Jeannette Jakus, Edward Heilman, R. Rivkah Isseroff, Daniel M. Siegel, Jared R. Jagdeo

**Affiliations:** 1 Department of Dermatology, SUNY Downstate Health Sciences University, Brooklyn, NY USA; 2 Dermatology Service, VA New York Harbor Healthcare System, Brooklyn, NY USA; 3 Statistical Design & Analysis Research Division, SUNY Downstate Health Sciences University, Brooklyn, NY USA; 4 Dermatology Service, Sacramento VA Medical Center, Mather, CA USA; 5 Department of Dermatology, University of California Davis, Sacramento, CA USA

**Keywords:** Light emitting diode, Red light, Phototherapy, Skin fibrosis, Wound healing, Scarring, Hypertrophic scar, Keloid, Surgery

## Abstract

**Background:**

Skin fibrosis is a significant global health problem that affects over 100 million people annually and has a profoundly negative impact on quality of life. Characterized by excessive fibroblast proliferation and collagen deposition, skin fibrosis underlies a wide spectrum of dermatologic conditions ranging from pathologic scars secondary to injury (e.g., burns, surgery, trauma) to immune-mediated diseases. Effective anti-scarring therapeutics remain an unmet need, underscoring the importance of developing novel approaches to treat and prevent skin fibrosis. Our in vitro data show that light emitting diode-red light (LED-RL) can modulate key cellular and molecular processes involved in skin fibrosis. In two phase I clinical trials (STARS 1 and STARS 2), we demonstrated the safety and tolerability of LED-RL at fluences of 160 J/cm^2^ up to 480 J/cm^2^ on normal human skin.

**Methods/design:**

CURES (Cutaneous Understanding of Red-light Efficacy on Scarring) is a dose-ranging, randomized, parallel group, split-face, single-blind, mock-controlled phase II study to evaluate the efficacy of LED-RL to limit post-surgical skin fibrosis in subjects undergoing elective mini-facelift surgery. Thirty subjects will be randomly allocated to three treatment groups to receive LED-RL phototherapy or temperature-matched mock irradiation (control) to either periauricular incision site at fluences of 160 J/cm^2^, 320 J/cm^2^, or 480 J/cm^2^. Starting one week post-surgery (postoperative days 4–8), treatments will be administered three times weekly for three consecutive weeks, followed by efficacy assessments at 30 days, 3 months, and 6 months. The primary endpoint is the difference in scar pliability between LED-RL-treated and control sites as determined by skin elasticity and induration measurements. Secondary outcomes include clinical and photographic evaluations of scars, 3D skin imaging analysis, histological and molecular analyses, and adverse events.

**Discussion:**

LED-RL is a therapeutic modality of increasing importance in dermatology, and has the potential to limit skin fibrosis clinically by decreasing dermal fibroblast activity and collagen production. The administration of LED-RL phototherapy in the early postoperative period may optimize wound healing and prevent excessive scarring. The results from this study may change the current treatment paradigm for fibrotic skin diseases and help to pioneer LED-RL as a safe, non-invasive, cost-effective, portable, at-home therapy for scars.

**Trial registration:**

ClinicalTrials.gov, NCT03795116. Registered on 20 December 2018.

**Electronic supplementary material:**

The online version of this article (10.1186/s13063-019-3546-6) contains supplementary material, which is available to authorized users.

## Background

Skin fibrosis, or scarring, represents an exuberant wound healing response following tissue damage due to stimuli such as infection, autoimmune reaction, and mechanical injury [1, 2]. Characterized by excessive fibroblast proliferation and collagen deposition in the dermis, skin fibrosis is the histopathologic hallmark of a wide spectrum of dermatologic diseases, including scleroderma, chronic graft-versus-host disease, and restrictive dermopathy [3, 4]. Localized skin fibrosis can also develop as a sequela of dermal injury (e.g., burns, surgery, trauma), manifested clinically as hypertrophic scars or keloids [5, 6].

Skin fibrosis is a significant global health problem with an estimated incidence of greater than 100 million persons affected per year in the developed world [7, 8]. Cutaneous scars have a profoundly negative impact on patients’ quality of life due to associated pain and pruritus, functional impairment, cosmetic disfigurement, and psychosocial distress [7, 9, 10]. As such, there is high demand for therapeutic modalities that prevent, reduce, or remove scars, as evidenced by an estimated $12 billion annual market for scar treatment in the US [11]. Despite the substantial socioeconomic burden associated with skin fibrosis, few effective and durable anti-scarring therapeutics are available, making scar treatment a major unmet medical need [12–14]. Furthermore, current scar management strategies may be invasive, cause undesirable side effects, or lack high-level evidence to support their use [12]. Therefore, it is important to research and develop novel approaches to treat and prevent skin fibrosis.

Visible light (400–700 nm) is ubiquitous in the environment and comprises 44% of total solar energy, yet its biological effects on the skin have not been fully elucidated [15, 16]. Visible light therapy delivered by light emitting diode (LED) devices is a therapeutic modality of increasing clinical importance in dermatology, as different wavelengths can alter skin physiology and produce beneficial effects such as in wound healing and skin rejuvenation [17–19]. Due to the significant advances in LED technology in recent years, LED phototherapy has become a valuable and effective treatment for a wide variety of medical and aesthetic conditions [20]. In 2017, members of the American Society for Dermatologic Surgery performed more than 3.2 million procedures using lasers, lights, and energy-based devices [21]. Furthermore, LED devices are commercially available and have US Food and Drug Administration (FDA) clearance for various dermatologic conditions including acne vulgaris and photoaging [19, 22]. Red light (630–700 nm) has the deepest tissue penetration depth of the visible light colors, reaching the entirety of the dermis where skin fibrosis occurs [18, 23, 24]. Recently published clinical observations indicate that red light in combination with other modalities, such as photosensitizers for photodynamic therapy, can decrease skin fibrosis [25–27].

According to our in vitro data, light emitting diode-red light (LED-RL) at high fluences (defined as equal to or greater than 160 J/cm^2^) can exert anti-fibrotic cutaneous effects by decreasing cellular proliferation, collagen production, and migration speed of human skin fibroblasts [28–31]. Prior to our studies on the anti-fibrotic properties of LED-RL, limited data existed regarding red light photobiomodulation of skin fibroblasts. In two phase I clinical trials, Safety Trial Assessing Red-light on Skin (STARS 1 and STARS 2), we demonstrated the safety and tolerability of LED-RL administered at fluences up to 480 J/cm^2^ on normal forearm skin in healthy individuals (*n* = 115 in both trials combined) [32]. Adverse events (AEs) included post-treatment erythema, hyperpigmentation, and localized bulla formation, all of which were mild and resolved without permanent sequelae (unpublished data).

CURES (Cutaneous Understanding of Red-light Efficacy on Scarring) is a phase II randomized controlled trial to evaluate the anti-fibrotic effects of LED-RL in subjects who will undergo elective mini-facelift surgery, using the periauricular skin incisions as the treatment sites. To our knowledge, no clinical trials have been performed to determine the safety and efficacy of LED-RL for skin fibrosis. Developing LED-RL as a modality for skin fibrosis would represent an important advance in scar therapy as it would offer many advantages over current therapeutic strategies. For example, LED-RL lacks serious systemic side effects associated with immunomodulatory agents, is non-invasive and avoids the need for painful injections with anti-fibrotic agents, requires no downtime for the patient, and does not generate procarcinogenic DNA damage associated with ultraviolet light therapy [31, 32]. The potential health impact of this study is significant, as successful demonstration of the clinical efficacy of LED-RL to limit post-surgical skin fibrosis may shift the current treatment paradigm for various types of scars. Therefore, we intend to study LED-RL as a standalone therapeutic modality for skin fibrosis, with the goal of pioneering LED-RL as a safe, cost-effective, non-invasive, portable, at-home treatment to reduce scarring.

## Methods/design

### Hypothesis

LED-RL phototherapy is a safe and effective therapeutic modality to limit post-surgical skin fibrosis.

### Primary outcome measure

Difference in quantitative scar pliability, as determined by skin elasticity and skin induration, between the treated and control incision sites.

### Secondary outcome measures

- Difference in the Patient and Observer Scar Assessment Scale (POSAS) scores between the treated and control incision sites
- Difference in the photograph-based Visual Analogue Scale (VAS) scores between the treated and control incision sites
- Difference in the objective measurements of key scar characteristics (collagen and water concentration, texture, volume, pigmentation, vascularity) between the treated and control incision sites
- Histological and molecular analyses of treated and control skin specimens
- Incidence of AEs

### Study setting and population

The study will be conducted at SUNY Downstate Medical Center. A total of 30 subjects will be enrolled. Individuals of any sex, ethnicity, and age who plan to undergo elective mini-facelift surgery may be eligible to participate in the study. The clinical research team will screen potential subjects (through interviews and physical examination) and determine eligibility according to the inclusion and exclusion criteria (Table 1). All mini-facelift procedures will be performed by the same surgeon. Prior to enrollment, a screening photosensitivity test will be conducted, wherein the potential subject will be exposed to LED-RL for 20 min on the non-dominant upper forearm, and then evaluated in clinic 24 h later for evidence of photosensitivity [32–34]. Criteria for photosensitivity include, but are not limited to, warmth, erythema, edema, rash, pain, or discomfort lasting more than 24 h. If no photosensitive reactions are noted, the subject will be eligible to enroll in the study.

**Table 1:** Eligibility criteria for the CURES trial

Inclusion criteria	Exclusion criteria
• Provision of written informed consent for all study procedures • Stated willingness to comply with all study procedures and availability for the duration of the study • Suitable candidate for elective mini-facelift surgery • Pass a screening photosensitivity test	• Current use of any photosensitizing medications • Light-sensitive conditions • Diabetes mellitus • Systemic lupus erythematosus • Current tobacco use • History of bleeding or coagulation disorder • Lax skin associated with genetic disorders • Open wounds on the face or neck • Fibrotic skin disease, pre-existing scar(s), or other skin conditions affecting the periauricular skin • History of surgery or procedure involving or affecting the periauricular skin within the past 6 months (e.g., prior facelift, fillers, laser therapy) • Tattoos that cover the proposed treatment sites on the periauricular skin • Any other medical condition(s) that could be compromised by exposure to the proposed treatment

### Study design

**Fig. 1:**
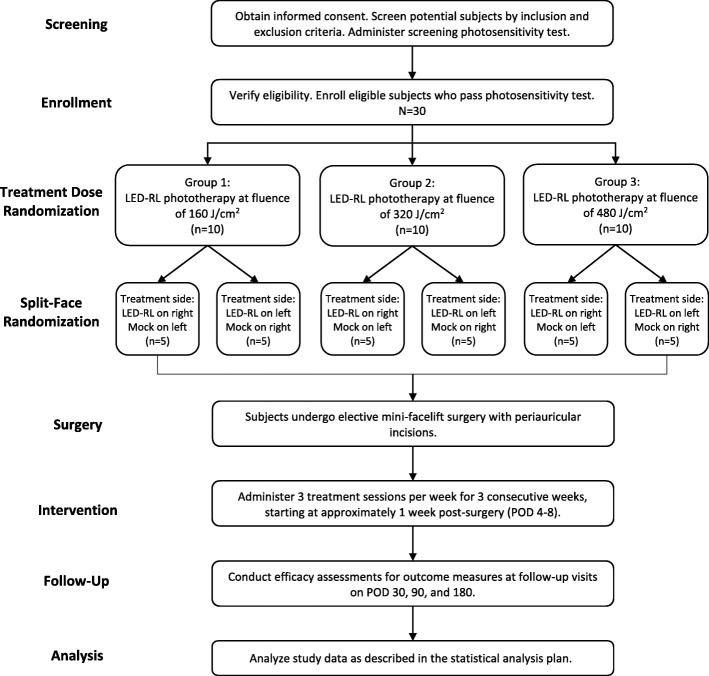
Study design for the CURES trial. *LED-RL* light emitting diode-red light, *POD* postoperative day

This is a dose-ranging, randomized, parallel group, split-face, single-blind, mock-controlled, phase II study to evaluate the efficacy of LED-RL in reducing skin fibrosis in subjects who will undergo elective mini-facelift surgery. Refer to Fig. 1 for a schematic of the study design. Starting one week after surgery (defined as postoperative days 4 to 8), subjects will receive LED-RL phototherapy to the periauricular skin incision sites. Beginning scar reduction therapy one week after surgery is a validated intervention time point for limiting surgical scars [35]. The maximum recommended starting dose of 160 J/cm^2^ is based on previously published maximum doses of LED-RL that demonstrated safety with no AEs in clinical studies [36, 37]. The highest dose to be tested is 480 J/cm^2^, which was found to be the maximum tolerated dose (MTD) in our phase I studies (unpublished data). The MTD is defined as the dose level below the dose producing unacceptable but reversible toxicity and is considered to be the upper limit of subject tolerance.

A total of 30 subjects will be randomly allocated to three treatment groups of ten subjects each to receive LED-RL phototherapy at the following fluences (doses): 160 J/cm^2^, 320 J/cm^2^, or 480 J/cm^2^. The treatment side (right face versus left face) will also be randomized to control for possible effects of uneven sun exposure. The untreated side will receive temperature-matched mock irradiation via a mock device that looks, sounds, and feels similar to the treatment device, but does not produce light. Subjects will receive their treatment sessions in-office three times weekly, a standard phototherapy regimen, for three consecutive weeks (i.e., a total of nine treatment sessions) [38–40]. This treatment schedule reflects the regimen implemented in our phase I studies [32]. Following the study intervention period, subjects will have follow-up assessments for outcome measures on approximately postoperative days (POD) 30, 90, and 180.

### Study devices

The LED-RL source is the Omnilux handheld LED system (GlobalMed Technologies, Glen Ellen, CA, USA). This LED-RL array device is commercially available as the Omnilux clear-U and Omnilux new-U, which are FDA-cleared for the treatment of facial acne and periorbital rhytides, respectively [33, 34, 41, 42]. The treatment device has a 4.7 cm × 6.1 cm rectangular array of LEDs and emits visible red light (633 ± 6 nm) at a power density of 360.2 W/m^2^ at room temperature and a distance of 10 mm from the target surface [30, 33, 34]. The mock therapy device is designed to sound, look, and feel identical to the LED-RL treatment device (i.e., has the same physical components and thermal output), except it does not emit visible red light [32]. The use of mock irradiation as a control ensures that any measured clinical effect is a result of LED-RL treatment and not due to ambient light, environment, or temperature [31]. The LED-RL treatment and mock therapy devices will be tracked using the manufacturer’s serial numbers.

### Study treatment

At the start of every treatment session, the subject’s periauricular skin on both sides of the face will be cleaned with alcohol pads and the treatment area will be outlined with a surgical marking pen, then photographed prior to device placement. The LED-RL treatment device and mock therapy device will be positioned in close contact with the periauricular skin (maximum distance of 10 mm from the skin surface), securely held in place for the duration of the treatment. LED-RL phototherapy and mock therapy will be administered simultaneously. Subjects will wear safety goggles during the treatment sessions, as recommended by Omnilux for comfort; there are no known ocular sequelae to LED-RL irradiation and the Omnilux devices have been tested to international standards to ensure that the outputs are safe for the eyes [33, 34]. The research coordinator will observe the treatment and monitor for any AEs or safety issues that may arise. Since the power density of LED-RL will remain constant across all treatment groups, the total fluence of LED-RL delivered depends on the exposure time to phototherapy [43]. The duration of the treatment administration for each treatment group is as follows:

- Group 1: LED-RL 160 J/cm^2^ and mock therapy (30 min)
- Group 2: LED-RL 320 J/cm^2^ and mock therapy (60 min)
- Group 3: LED-RL 480 J/cm^2^ and mock therapy (90 min)

### Concomitant therapy

A review of concomitant medications will be performed at each study visit. The concurrent use of photosensitizing medications is a contraindication to LED phototherapy [18, 44]. Therefore, during the three-week intervention period, the following medications that are known to have photosensitizing properties will be prohibited: amiodarone, azathioprine, chlorpromazine, gold, griseofulvin, isotretinoin, lithium, melatonin, methotrexate, phenothiazine antipsychotics, tetracycline antibiotics, and quinolone derivative antibiotics. For the entire duration of the study, subjects will be asked to avoid scar treatments to both periauricular incision sites (with the exception of topical agents recommended for routine postoperative wound care), as to not confound the results of the study. Scar treatments include, but are not limited to, silicone gels/sheets, intralesional corticosteroids, 5-fluorouracil, laser therapy, radiotherapy, cryotherapy, bleomycin, mitomycin C, imiquimod, pressure therapy, adhesive microporous hypoallergenic paper tape, onion extract, massage therapy, over-the-counter topical emollients for scars, laser therapy, and surgical revision [13].

### Efficacy assessments

Efficacy assessments for outcome measures will be conducted at baseline (i.e., at the first treatment session, prior to initiation of LED-RL phototherapy) and at follow-up visits on approximately POD 30 (1 month), POD 90 (3 months), and POD 180 (6 months). The evaluations at multiple time points will allow comparison of the difference between treated and control sites over time as the post-surgical scars heal and mature.

#### Primary outcome measures

The primary endpoint is the difference in quantitative scar pliability between the treated and control incision sites at 1 month, 3 months, and 6 months after surgery. The time point of primary interest is the final assessment at 6 months. Skin elasticity and skin induration are two separate indices for scar pliability and will be objectively measured using the ElastiMeter and the SkinFibroMeter (Delfin Technologies, Kuopio, Finland), respectively, which have been shown to be accurate and reliable to assess skin fibrosis [45–47]. These non-invasive instruments have an indenter and force sensors to measure the force resisting vertical deformation of the skin surface [48]. Scar tissue is thicker than normal skin and therefore is expected to be firmer and less compliant (i.e., more stiff) [49, 50]. The measurements will be taken at the midpoint of width and length of each scar to ensure that recurrent measurements will be in the same anatomic location.

#### Secondary outcome measures

Standardized scar evaluations using the POSAS will be performed by the same investigator (who is blinded to the treatment or control site) in conjunction with the subject at baseline and each follow-up visit. The POSAS is a validated tool to evaluate post-surgical linear scars and is commonly used in clinical trials to assess scar quality in response to treatment [51–54]. The two subscales of the POSAS each consist of six items rated from 1 to 10, where 1 is “normal skin” and 10 is the “worst imaginable scar”. The observer (i.e., investigator) evaluates scar vascularity, pigmentation, thickness, relief, pliability, and surface area while the patient assesses pain, itching, color, stiffness, thickness, and irregularity. The scores of each of the six items are summed for a total score (range 6 to 60). Importantly, the POSAS incorporates the patient’s judgment of scar appearance and includes subjective symptoms of pain and pruritus, allowing for a more comprehensive evaluation of treatment response and patient-reported outcomes [50, 55].

Standardized digital photographs of the incision sites will be taken at baseline and each follow-up visit. The scar images will be rated by two independent, blinded dermatologists using a visual analogue scale (VAS). The VAS is presented as a 10-cm horizontal line, where the extreme ends of 0 indicates “normal skin” and 10 corresponds to the “worst possible scar”, for each of the following scar attributes: pigmentation, vascularity, observer comfort, contour, and overall severity [49, 56]. The evaluators will also be presented with paired scar images (i.e., LED-RL-treated scar paired with its matched control site) and asked to rank which scar appears better. The photographic-based VAS scoring and ranking system has been shown to be consistent, reliable, and valid for linear scar assessment [57].

Secondary outcome measures will also include objective measurements of important scar characteristics such as physiological properties, dimensions, and color. A non-invasive, handheld diffuse reflectance probe will be used to measure collagen and water concentration in the dermis at the midpoint of the scars [58]. A 3D digital imaging system will be used to construct 3D images of the scars for skin profilometry and colorimetric analyses, including quantitative measurements of texture, pigmentation, vascularity, surface area, and tissue volume [59–61].

#### Histological and molecular studies

The histological and molecular changes that occur in vivo in response to LED-RL phototherapy will be evaluated by examination of pre- and post-treated skin tissue. Skin specimens will be obtained via optional 2 mm punch biopsy on POD 0 (from excised periauricular skin on the day of surgery) and POD 30 (post-treatment incision sites at the first follow-up visit). Subjects will have the option to decline biopsies and remain in the study. Histological examination of skin specimens will be conducted to quantify the number of Ki-67 positive fibroblasts and to assess collagen content [62]. High-throughput assays will be performed, including RNA sequencing, microRNA arrays, and quantitative real-time PCR, to screen for molecular effects (e.g., changes in gene expression) associated with LED-RL exposure.

### Safety assessment

Subjects will be provided with a daily diary to record any AEs experienced during the 3-week intervention period, and will also be called weekly to monitor for AEs. Treatment sessions will be monitored closely for the occurrence of any safety issues or AEs, as reported by the subject or observed by the clinical research team. The incision sites will also be photographed before and after each treatment administration for visual documentation of the subject’s initial presentation and any subsequent changes. All AEs will be documented in the appropriate case report form (CRF) with details including the event description, time of onset, severity, relationship to the study intervention, and outcome. An AE is defined as any untoward medical occurrence associated with the use of the study intervention, whether or not considered to be causally related [63]. All AEs will be followed for outcome information until adequate resolution or stabilization. Common expected post-treatment side effects, including warmth, redness, and swelling, are expected to be transient (i.e., last less than 24 h) and will be recorded, but will not be considered AEs in safety data reports [32]. The study may be halted or a subject may be withdrawn from the study if necessary for safety reasons, such as the occurrence of serious AEs (e.g., second-degree or higher skin burning, severe blistering, persistent swelling or pain, ulceration, change in sensation, muscle weakness, worsening of scar).

### Randomization

All subjects will be assigned to the treatment group and LED-RL treatment side simultaneously as they are enrolled by the research coordinator. A block randomization scheme will be used to ensure balanced allocation of subjects to the study arms, such that ten subjects are assigned to each treatment group (i.e., allocation ratio 1:1:1) and such that equal numbers receive LED-RL to the right face and left face (Fig. 1). The block sizes will not be disclosed to ensure concealment. The allocation sequence will be generated by a research assistant not involved in enrolling participants or assigning interventions, using a computer-based random number generator [64]. Individual assignments to Group 1 (LED-RL 160 J/cm^2^), Group 2 (LED-RL 320 J/cm^2^), or Group 3 (LED-RL 480 J/cm^2^) as well as the LED-RL treatment side (right versus left) will be concealed together in sequentially numbered, opaque, sealed envelopes until the time of enrollment.

### Blinding

Subjects will be blinded to the study intervention (LED-RL phototherapy versus mock therapy) as the treatment area is outside of the range of view and the study devices are indistinguishable. Clinicians involved in subjective efficacy assessments will be also be blinded. This includes the investigator performing the POSAS, the two independent dermatologists evaluating the scar photographs, and the dermatopathologist examining the skin specimens. The principal investigator (PI), along with the research coordinator who administers the LED-RL phototherapy, will be aware of each subject’s treatment assignment. Unblinding may occur at the discretion of the PI in exceptional circumstances, such as when knowledge of the study intervention is needed to treat a serious AE.

### Time frame

This study is designed to conclude in 12 months, which includes subject screening and enrollment, study intervention, follow-up for efficacy assessments, and data analysis. The full schedule of clinical trial activities is available in accordance with SPIRIT guidelines (Additional file 1).

### Sample size justification

This clinical trial is intended to be a pilot study to obtain estimates of feasibility and outcome variability, as there is a paucity of background data in the literature. These precise estimates will aid in the planning of a larger, sufficiently powered efficacy trial. We estimate that a difference of 15% in scar pliability will be clinically meaningful, based on the minimum decrease in fibroblast number in response to LED-RL irradiation from our in vitro data [29]. A sample size of 30 subjects (with the split-face, intra-individual comparison design) will allow for a precise estimate of the variance in scar pliability change in this population.

### Statistical analysis

SAS version 9.4 statistical package (SAS Institute, Cary, NC, USA) will be used for intention-to-treat analysis (for all available data) and per-protocol analysis (for subjects with complete data). The primary outcome measures will be used separately as dependent variables (DVs) in mixed linear models. Fixed factors in each model will be treatment group, whether treated, side of face (left versus right), and time (three follow-up assessments post-baseline). Baseline DV measure will be introduced as a scored covariate, with subject identification as a random factor. The Akaike information criterion will be used to assess what intra-subject covariance structure might be optimal. Tests of interaction among fixed factors will be conducted and the utility of polynomial terms in the baseline DV investigated. Model residuals will be examined for skew and for outliers; the DV will be power-transformed if necessary to maximize normality of residuals. Secondary outcomes will be presented as descriptive statistics tabulated by whether treated, treatment group, and time. No inferential analyses will be conducted in order to minimize the multiple testing problem.

Summary statistics of safety data will be presented as number of subjects who experienced AEs in each treatment group and a breakdown of the event types. Descriptive statistics of relevant subject characteristics (age, gender, race, and ethnicity) of the study population will be tabulated by treatment group. Continuous variables will be presented as median and range. Categorical variables will be presented as proportions. Significance tests will not be applied. To determine if any differential effects of LED-RL are associated with demographics, a linear regression or robust linear regression with adjustment for treatment group effect on scar pliability will be performed.

### Data management and monitoring

All individual subject data required by the study protocol will be recorded in the appropriate CRFs as well as in a 21 CFR Part 11-compliant data capture system, in accordance with the International Conference on Harmonisation (ICH) Good Clinical Practice (GCP) guidelines. All source documents will be de-identified, securely maintained, and protected for confidentiality in accordance with the SUNY Downstate Institutional Review Board (IRB) policies. Research records will be accessible to the research coordinator, investigators, and data/safety monitoring personnel as required.

A data monitoring committee (DMC) composed of three board-certified dermatologists, independent from the study conduct and free of conflict of interest, will be responsible for safety oversight and clinical monitoring. The DMC will convene on a quarterly basis to review any AEs and safety issues. All AEs and serious AEs will be reported to the IRB and DMC within the timelines specified in the SUNY Downstate IRB policy for human research protections. The PI will have access to interim results and be able to make the final decision to terminate the study.

## Discussion

To our knowledge, no prior clinical trials have been performed to determine the safety and efficacy of LED-RL phototherapy for the treatment or prevention of skin fibrosis. This study may provide important data on LED-RL phototherapy as a therapeutic modality for post-surgical skin scarring, and help to facilitate future clinical trials to further evaluate the efficacy of LED-RL in comparison to existing anti-fibrotic therapeutics for various types of tissue fibrosis.

While scar formation is an expected outcome of wound healing after cutaneous injury, it can range from faint scarring to severe thickening and tightening of the skin [65]. Scar prevention is a key consideration in postoperative wound management, as scarring has significant aesthetic and functional consequences for patients [66, 67]. While scar revision (i.e., treatment of existing scars) has been the mainstay of therapy, recent advances in the understanding of wound healing mechanisms have inspired innovative strategies to prevent scar formation [68–70]. As in vitro data show that LED-RL can attenuate profibrotic cellular processes that contribute to skin fibrosis, LED-RL is a promising strategy to minimize scar formation after surgery [29, 31]. In this study, LED-RL phototherapy will be initiated within one week post-surgery (defined as POD 4–8), coinciding with the early proliferation phase of wound healing, which will help to answer important questions about the impact of intervention time on final scar outcomes [71–73].

The methodology described in this protocol offers several advantages compared to other clinical trials that evaluate scar management strategies. The split-face study design allows each subject to serve as his or her own control, such that comparisons of clinical efficacy between treated and control scars are within-subject (i.e., intra-individual). Therefore, any measured changes in scar characteristics can be attributed to the treatment, eliminating the confounding factor of inter-individual differences in wound healing. It is important to note that in the prospective evaluation of scar reduction therapy, it is assumed that, if left untreated, the bilateral facelift incisions would heal with identical scars. Since wound healing and scar formation are influenced by many variables, a self-controlled study design is favorable to allow detection of true treatment effects. Furthermore, the treated side of the face will be randomized to account for possible differences in the laterality of photodamaged skin (e.g., asymmetry of sun exposure in automobile drivers) [74].

A dose-ranging study design is implemented as the safety of LED-RL phototherapy in a facelift scar model may differ from the safety in normal skin. For example, LED-RL fluences determined to be safe in normal forearm skin (the treatment site in STARS 1 and STARS 2) may have different effects on the face, as physiological properties of skin vary depending on anatomic location [75, 76]. Thus, the MTD established in our phase I studies serves as the upper limit of treatment dose in this study.

This study may have several potential limitations. There may be a bias in age toward middle-aged and elderly individuals, as the majority of facelift patients are over the age of 50 years (mean 59 years) [77, 78]. Increased age is associated with reduced collagen turnover due to a decrease in fibroblast collagen synthesis; therefore, the penetration of LED-RL may be affected and certain fluences that result in AEs may vary among different age groups [79, 80]. Furthermore, since cell turnover is a major contributor to the development of scar tissue in a healing wound, elderly individuals tend to have better outcomes for scar cosmesis and are less susceptible to exuberant, pathologic scarring [81–84].

Limited data exist regarding the cutaneous effects of LED-RL on different skin types and ethnicities. Based on the safety data generated in our phase I studies, we hypothesized that LED-RL may exhibit differential biologic effects depending on ethnicity, with skin of color individuals being more photosensitive to LED-RL compared to Caucasian non-Hispanic individuals. To further explore this observation, this study will test the MTD of 480 J/cm^2^ in all subjects regardless of skin type, and subgroup analyses will be performed to assess any differences in AEs based on demographics.

There is a large unmet need for innovative therapeutic strategies to manage skin fibrosis, such as excessive scar formation after surgery [85]. Despite the substantial healthcare burden of skin fibrosis, there is no “gold standard” or universally effective scar therapy, and current treatment options have limited clinical efficacy [12, 67, 86]. Therefore, successful demonstration of the efficacy of LED-RL phototherapy in limiting skin fibrosis may revolutionize the current treatment paradigm for fibrotic skin diseases. Future studies may extend beyond scar prevention and investigate the use of LED-RL to treat existing scars.

## Trial status

The final protocol version is 4.1 and dated 4 April 2019. This study began recruiting subjects on 18 March 2019 at SUNY Downstate Medical Center, Brooklyn, NY, USA. We expect all participants to be enrolled by 1 July 2019.

## Additional files

Additional file 1: Study participant schedule of activities and procedures. (DOCX 13 kb)

Additional file 2: SPIRIT Checklist. (PDF 1055 kb)

## Data Availability

The datasets generated during this study will be available from the corresponding author on reasonable request. On completion of the trial, the data will be analyzed and a final report of study findings will be prepared for submission to a scientific journal.

## Abbreviations

AE: Adverse event
CRF: Case report form
DV: Dependent variable
FDA: Food and Drug Administration
GCP: Good Clinical Practice
ICH: International Conference on Harmonisation
IRB: Institutional Review Board
LED: Light emitting diode
LED-RL: Light emitting diode-red light
MTD: Maximum tolerated dose
PI: Principal investigator
POD: Postoperative day
POSAS: Patient and Observer Scar Assessment Scale
VAS: Visual analog scale
