# Supplementary material for: A dose-ranging, parallel group, split-face, single-blind phase II study of light emitting diode-red light (LED-RL) for skin scarring prevention: study protocol for a randomized controlled trial
Source: Trials. 2019 Jul 15;20:432. doi: 10.1186/s13063-019-3546-6 (PMC6631489; doi:10.1186/s13063-019-3546-6)
Supplement: Supplementary file 1 — Study participant schedule of activities and procedures. (DOCX 13 kb) [file 13063_2019_3546_MOESM1_ESM.docx]

|  | Screening within 6 weeks prior to surgery | Enrollment at 24 hours after screening | Baseline/day of surgery  (Visit 1) | Treatment #1  (Visit 2) | Treatment #2  (Visit 3) | Treatment #3  (Visit 4) | Treatment #4  (Visit 5) | Treatment #5  (Visit 6) | Treatment #6  (Visit 7) | Treatment #7  (Visit 8) | Treatment #8  (Visit 9) | Treatment #9  (Visit 10) | Follow-up at POD 30 (Visit 11) | Follow-up at POD 90 (Visit 12) | Final study visit at POD 180 (Visit 13) |
| --- | --- | --- | --- | --- | --- | --- | --- | --- | --- | --- | --- | --- | --- | --- | --- |
| **Screening & Enrollment** |  | | | | | | | | | | | | | | |
| Eligibility screen | X |  |  |  |  |  |  |  |  |  |  |  |  |  |  |
| Informed consent | X |  |  |  |  |  |  |  |  |  |  |  |  |  |  |
| Screening photosensitivity test (administration) | X |  |  |  |  |  |  |  |  |  |  |  |  |  |  |
| Screening photosensitivity test (evaluation) |  | X |  |  |  |  |  |  |  |  |  |  |  |  |  |
| Randomization |  | X |  |  |  |  |  |  |  |  |  |  |  |  |  |
| Mini-facelift surgery |  |  | X |  |  |  |  |  |  |  |  |  |  |  |  |
| **Study Intervention** |  | | | | | | | | | | | | | | |
| LED-RL phototherapy |  |  |  | X | X | X | X | X | X | X | X | X |  |  |  |
| Mock therapy |  |  |  | X | X | X | X | X | X | X | X | X |  |  |  |
| **Efficacy Assessments** |  | | | | | | | | | | | | | | |
| Adverse events |  |  |  | X | X | X | X | X | X | X | X | X | X | X | X |
| Digital photography |  |  | X | X | X | X | X | X | X | X | X | X | X | X | X |
| Skin biopsy (optional) |  |  | X |  |  |  |  |  |  |  |  |  | X |  |  |
| OCT |  |  |  | X |  |  |  |  |  |  |  |  | X | X | X |
| Skin elasticity and induration measurements |  |  |  | X |  |  |  |  |  |  |  |  | X | X | X |
| Collagen and water concentration measurements |  |  |  | X |  |  |  |  |  |  |  |  | X | X | X |
| Skin imaging analysis |  |  |  | X |  |  |  |  |  |  |  |  | X | X | X |
| POSAS |  |  |  | X |  |  |  |  |  |  |  |  | X | X | X |
